# Late-maturity α-amylase (LMA): exploring the underlying mechanisms and end-use quality effects in wheat

**DOI:** 10.1007/s00425-021-03749-3

**Published:** 2021-11-27

**Authors:** Ashley E. Cannon, Elliott J. Marston, Alecia M. Kiszonas, Amber L. Hauvermale, Deven R. See

**Affiliations:** 1 Wheat Health, Genetics, and Quality Research Unit, USDA Agricultural Research Service, Pullman, WA USA; 2 Department of Crop and Soil Sciences, Washington State University, Pullman, WA USA; 3 Department of Plant Pathology, Washington State University, Pullman, WA USA

**Keywords:** Wheat, End-use quality, Falling numbers, Late-maturity α-amylase, Genetics, Environmental impact

## Abstract

**Main conclusion:**

A comprehensive understanding of LMA from the underlying molecular aspects to the end-use quality effects will greatly benefit the global wheat industry and those whose livelihoods depend upon it.

**Abstract:**

Late-maturity α-amylase (LMA) leads to the expression and protein accumulation of high pI α-amylases during late grain development. This α-amylase is maintained through harvest and leads to an unacceptable low falling number (FN), the wheat industry’s standard measure for predicting end-use quality. Unfortunately, low FN leads to significant financial losses for growers. As a result, wheat researchers are working to understand and eliminate LMA from wheat breeding programs, with research aims that include unraveling the genetic, biochemical, and physiological mechanisms that lead to LMA expression. In addition, cereal chemists and quality scientists are working to determine if and how LMA-affected grain impacts end-use quality. This review is a comprehensive overview of studies focused on LMA and includes open questions and future directions.

## Introduction

Wheat is one of the most important global food crops in temperate zones, and the demand for this crop is continuing to grow. In addition to serving as a major source of starch and energy in traditional western diets, wheat crops are economically important worldwide. The Hagberg–Perten falling number (FN) test is the industry’s standard to gauge the predicted end-use quality of wheat flour (Hagberg 1960, 1961; Perten 1964). This test measures the functional integrity of a whole meal slurry derived from ground wheat grains and water by monitoring the gelling capacity of the slurry that is formed by mixing and heating the wheat flour and water mixture. When starch is degraded by hydrolytic enzymes, the gelling capacity of this mixture decreases, leading to a low FN and a decrease in end-use quality (Hagberg 1960, 1961; Perten 1964). In the United States Pacific Northwest, discounts are applied for every 25 s below 300 s (Steber 2017). Furthermore, if low FN is detected in Australia, there is a monetary loss of $AUS20-50 per ton (Newberry et al. 2018). These discounts contribute additively to the financial hardships already experienced by growers.

The potential causes of low FN in wheat include pre-harvest sprouting (PHS), late-maturity α-amylase (LMA), and retained pericarp α-amylase (Lunn et al. 2001; Mares and Mrva 2008, 2014). PHS is the germination of mature grain in the spike prior to harvest as a result of continuous rain and high humidity (Mares 1993; Mares and Mrva 2014). During sprouting, hydrolytic enzymes are initially produced in the epithelial cells of the scutellum, but as germination progresses, the aleurone layer takes over the role of enzyme synthesis and secretion (Fincher 1989). The secreted enzymes are transported to the endosperm where they degrade starch, proteins, lipids, and cell wall materials. The products of the germination process are transported to the scutellum and provide the energy and building blocks needed for germination (Fincher 1989). During sprouting, these degradative processes decrease the quality of wheat flour when used to make breads, cakes, noodles, and other cereal-based products (Olaerts and Courtin 2018). LMA leads to the production of high isoelectric point (high pI) α-amylases in the aleurone as a result of a temperature shock during mid-grain development or prolonged cold throughout grain development (Mares and Mrva 2008; Barrero et al. 2013, 2020; Derkx and Mares 2020). During LMA, *α-Amylase1* (*α-Amy1*) genes are expressed, resulting in the synthesis of enzymes that are retained in the grain until harvest and lead to unacceptably low FN (Mrva and Mares 1999; Barrero et al. 2013; Cheng et al. 2014; Mieog et al. 2017). In contrast to PHS, where there is a gradient of α-amylase activity in the grain that is driven by an embryo-derived signal, LMA leads to a random distribution of α-amylase activity throughout the grain (Mrva and Mares 1996a; Mrva et al. 2006). Retained pericarp α-amylase, a rarer cause of low FN, is caused by low temperatures that prevent the degradation of low pI α-amylases in the pericarp during early grain development (Lunn et al. 2001).

In recent years, studies focused on the underlying genetic, biochemical, and physiological mechanisms that lead to LMA have increased (Kondhare et al. 2012, 2013, 2014; Barrero et al. 2013, 2020; Cheng et al. 2014; Derkx and Mares 2020; Derkx et al. 2021; Liu et al. 2021). Collectively, LMA studies have shown that this condition is a consequence of genotype, environment, and grain developmental stage (Mares and Mrva 2014; Derkx and Mares 2020; Derkx et al. 2021). In addition, the level of expression of pre-maturity, high pI α-amylase is variable and hard to predict. As a result, this condition has been a major challenge to researchers, breeders, and members of the wheat industry, and after decades of work, our understanding of this phenomenon is still incomplete. In addition, as described above, LMA may compromise the ability to produce high-quality baked goods from wheat flour (Kiszonas et al. 2018; Newberry et al. 2018). Here, we describe studies that identify the underlying mechanisms that lead to LMA in wheat, describe the effects of this condition on end-use quality, and identify open questions and topics for future studies.

## Genetic mechanisms of LMA

As early as the 1960s, researchers noticed that the UK cultivar, Professeur Marchal, produced α-amylase in the absence of sprouting (Bingham and Whitmore 1966; Gale and Marshall 1975; Gale et al. 1987). Mohler et al. (2014) suggested that Maris Huntsman, a descendant of Professeur Marchal, was the initial source of LMA within the UK germplasm. Australian researchers did not identify LMA-susceptible germplasm in the Australian breeding program until the 1990s. Research on Australian germplasm was largely focused on the LMA-susceptible cultivars, Spica and Lerma-52, but later research involved extensive testing of the germplasm collection, including within the pedigrees of Spica and Lerma-52 (Mrva and Mares 1996b; Mares and Mrva 2008). The source of Spica’s genetic susceptibility to LMA has not been identified, but Mares and Mrva suggested that Lerma-52 inherited the tendency to produce excess post-harvest α-amylase from the recurrent parent, Mentana (2008). The cultivar Squarehead appears twice in the pedigree of Mentana. Squarehead and descendants also appear multiple times in the pedigree of Professeur Marchal, making Squarehead a common ancestor of LMA-susceptible lines in the UK, German, and Australian germplasm (Dobrotvorskiy et al. 2021).

Recent studies examining the prevalence of LMA susceptibility within germplasm collections suggests that LMA may be more common than originally thought. Liu et al. (2021) phenotyped germplasm from ten geographical regions of North America, 251 hard spring lines in total, and identified reproducible LMA resistance in 27% of the lines. A substantial number of LMA-susceptible lines have also been identified in germplasm from Australia, Japan, Canada, South Africa, China, Mexico, Germany, and the UK (Mares and Mrva 2008; Mohler et al. 2014). In addition, when Mrva et al. (2009) phenotyped 253 synthetic hexaploid varieties and more than 300 derived synthetic lines, more than 85% of the synthetic lines provided by CIMMYT were found to be LMA susceptible. Synthetics were grouped according to their genotypes at the LMA-associated QTL on 3B and 7B, which revealed an association between genotype and the durum parents of the synthetic lines. Germplasm developed by CIMMYT has been disseminated widely across the globe, but the incidence of LMA in durum lines, as well as in breeding programs with low levels of CIMMYT material, suggests that LMA susceptibility may be more common than LMA resistance and may not have been introduced to breeding programs by a single source as has been previously suggested.

Studies have demonstrated that LMA is a multi-genic trait, with associated QTL across all three genomes that appear to be independently effective and additive in their contribution to the LMA phenotype (Mrva and Mares 2002; Mares and Mrva 2008; Derkx et al. 2021). At this time, the major QTL located on the long arm of 7B explains 31–42% of LMA phenotypic variance (Mrva and Mares 2001a; Derkx et al. 2021). The 7B QTL has been confirmed in nine publications and identified in germplasm from genetically diverse breeding programs (Mrva and Mares 2001a; Mrva et al. 2009; Emebiri et al. 2010; Mohler et al. 2014; Zhang et al. 2014; Martinez et al. 2018; Börner et al. 2018; Derkx et al. 2021). Fifteen minor QTL have also been identified. These LMA-associated QTL have been mapped to all seven chromosome groups and across the three genomes (Table 1).

**Table 1:** LMA-associated QTL with proposed candidate genes and publications identifying specific QTL

LMA QTL	Candidate genes	Publications
1AS	–	Verbyla and Cullis (2012), Liu et al. (2021)
1BL/1RS	–	Mohler et al. (2014)
2DL	–	Tan et al. (2010), Mohler et al. (2014)
3A	Homologous gene to 3B QTL	Tan et al. (2010), Verbyla and Cullis (2012), Liu et al. (2021)
3B	Associated with a *Triticum aestivum* Boron transporter2 mRNA	Mrva and Mares (2001a), Verbyla and Cullis (2012), Zhang et al. (2014), Liu et al. (2021)
3D	Homologous gene to 3B QTL	Tan et al. (2010), Verbyla and Cullis (2012)
4A	β*-Amy-A1*	Mohler et al. (2014), Bevan (2020)
4B	*Rht1*	Mrva and Mares (2001a), Mrva et al. (2009), Tan et al. (2010), Verbyla and Cullis (2012)
4D	*Rht2*	Tan et al. (2010), Verbyla and Cullis (2012), Mohler et al. (2014), Börner et al. (2018)
5A	α*-Amy-A3*	Bevan (2020)
5BL	*α-Amy-B3*	Tan et al. (2010), Verbyla and Cullis (2012), Mohler et al. (2014)
5DS	–	Tan et al. (2010), Verbyla and Cullis (2012)
6A	–	Mohler et al. (2014)
6B	*α-Amy-B1*	Emebiri et al. (2010), Mohler et al. (2014), Bevan (2020), Liu et al. (2021)
7A	–	Mrva and Mares (2001a), Mohler et al. (2014), Bevan (2020), Liu et al. (2021)
7BL (*LMA-1)*	ent-copalyl diphosphate synthase	Mrva and Mares (2001a), Mrva et al. (2009), Emebiri et al. (2010), Mohler et al. (2014), Zhang et al. (2014), Martinez et al. (2018), Börner et al. (2018), Derkx et al. (2021)
7D	–	Liu et al. (2021)

The 7B QTL is the first to be mapped to a single candidate gene. Designated *LMA-1*, the proposed gene encodes a putative ent-copalyl diphosphate synthase (CPS), which in other plant species is involved in gibberellin (GA) biosynthesis (Derkx et al. 2021). When gene sequences were examined, LMA-susceptible varieties carried a fully functional protein encoding gene sequence. However, LMA-resistant varieties carried alleles that led to premature stops or mutations that could result in a functionally altered version of the encoded protein. The *LMA-1* locus had nine haplotypes which had varying effects on the LMA phenotype (Derkx et al. 2021).

The minor QTL associated with LMA do not have candidate genes proposed, though several QTL appear to co-locate with known genes. The QTL on 4B and 4D co-locate the two most commonly utilized reduced height genes in hexaploid wheat, *Rht1(Rht-B1b)* and *Rht2 (Rht-D1b)* (Mrva and Mares 2001a; Mrva et al. 2009; Tan et al. 2010; Verbyla and Cullis 2012; Mohler et al. 2014; Börner et al. 2018). There are currently 24 genes known to reduce plant height in wheat and they can be grouped into two categories: GA sensitive and GA insensitive (Zhang et al. 2021). *Rht1* and *Rht2* are GA insensitive, have a semi-dwarf phenotype, and encode DELLA proteins, which inhibit downstream signaling of GA. GA-sensitive *Rht* genes, such as *Rht8, Rht13, and Rht18,* are characterized by a deficiency of endogenous GA rather than the inability to sense GA. Studies have not yet identified the mechanism behind reduced height in all GA-sensitive *Rht* genes, though research into their agronomic advantages suggests that the mechanisms are associated with a plant’s ability to tolerate abiotic stress (Ford et al. 2018; Tang et al. 2020; Yan et al. 2020; Mohan et al. 2021). *Rht18* appears to reduce levels of bioavailable GA through upregulated expression of *GA2oxA9,* a GA-2 oxidase that metabolizes intermediates of bioactive GA into inactive products (Ford et al. 2018).

The relationship between endogenous GA levels, GA sensitivity of DELLA proteins, and cold shock on the level of alpha-amylase in LMA-sensitive wheat varieties needs further study. Identifying and developing research lines which are fixed at loci of known, but less common, GA-insensitive and GA-sensitive genes should be a long-term research goal for the larger scientific community. *Rht1* and *Rht2* and their alleles have been well characterized in the literature since the Green Revolution, but the ability to directly sequence genes allows us to investigate the influence of single base pair mutations within an allele of a single gene. The *Rht-B1c* allele of *Rht1* is characterized by a 2 kb insertion that results in the addition of 30 amino acids that are in frame with the adjacent DELLA motif (Van De Velde et al. 2017). This leads to a disruption of the binding site between GID1 (a GA receptor) and the DELLA-B1c protein, complete dwarfism, and strong dormancy. The spring wheat cultivar Maringa was used to create intragenic alleles of the *Rht-B1c* gene, 14 of which displayed a consistent plant phenotype of either tall or semi-dwarf and were termed overgrowth genes (*ovg).* By backcrossing the *ovg* genes into diverse backgrounds and growing the lines in multiple environments (greenhouse and field experiments in Germany and Australia), consistent effects on yield, grain quality, and vegetative organ growth were observed. The design of this study along with direct sequencing allowed the genetic effects of mutations within the *Rht-B1c* gene to be separated from the noise of environment and genotype interactions. Mutations that occurred at either the N- or the C-terminus of the DELLA protein had significantly different yield and vegetative organ growth that was consistent across genotypes and environments. The *ovg* genes may provide a novel way of investigating LMA’s relationship with GA that is more nuanced than in previous studies, which focused on LMA’s interaction with *Rht1* and *Rht 2.*

It is also possible that novel *Rht* genes or alleles exist in the D genome. Crosses between LMA-resistant, semi-dwarf hexaploid wheat and synthetic hexaploid wheat varieties resulted in nearly 25% of the progeny displaying a tall phenotype and a high instance of LMA expression. This result suggested that GA insensitivity may be the sole source of the requirement for a cold shock to induce LMA in semi-dwarf lines (Tan et al. 2010). The link between LMA expression and GA-insensitive dwarfing genes was identified in the first studies describing LMA (Mrva and Mares 1996b). Further studies showed the presence of GA-insensitive, dwarfing genes reduced the constitutive expression of α-amylases associated with LMA (as observed in tall, *rht* cultivars) to stochastic expression that is induced by the presence of a cold temperature shock (Mrva and Mares 2001b; Tan et al. 2010). Alternatively, in synthetic hexaploid wheat varieties, created by the artificial hybridization of *Triticum durum* (AABB) and *Aegilops tauschii* (DD), the presence of GA-insensitive dwarfing genes does not reduce the incidence of LMA. The GA-insensitive dwarfing genes from the durum parent appear to be suppressed in the presence of the D genome, with most synthetic hexaploid wheat lines exhibiting tall phenotypes. This suppression of GA insensitivity conferred by the dwarfing genes resulted in more than 85% of the surveyed synthetic hexaploids exhibiting LMA susceptibility (Mrva et al. 2009).

QTL located on 4A, 5A, 5B, and 6B appear to co-locate with known α-amylase or b-amylase genes (Mohler et al. 2014; Bevan 2020). Gene copies of *Amy1*, *Amy2,* and *Amy3* map to group 6, group 7, and group 5 chromosomes, respectively (Zhang and Li 2017). *Amy1, Amy2,* and *Amy3* produce α-amylase enzymes that play unique roles at different stages of grain development, maturation, and germination in wheat grains. These different alleles may lead to differences in total α-amylase accumulation, but do not necessarily confer LMA susceptibility or resistance. The QTL on 3A, 3B, and 3D appear to be associated with a boron transporter gene (Zhang et al. 2014). Additionally, the 7B QTL is influenced by its proximity to the *Bo1* locus that encodes a boron transporter protein. Boron is an essential micronutrient for plants and plays an important role in plant cell wall structure, sugar mobilization, and formation of viable reproductive structures (Bell 2017; Brdar-Jokanović 2020). In wheat, boron uptake efficiency can affect the timing of anthesis, grain number per spike, dry grain weight, and yield (Rerkasem and Jamjod 2004; Tahir et al. 2009; Shaaban 2010). If a boron-deficient or boron toxic state exacerbates the severity or incidence of LMA, the presence or absence of the *Bo1* gene may explain a portion of both genetic and environmental variance within an LMA phenotype. Studies have shown that the uptake and transportation of boron is reduced under cold stress, and that the ability to move boron under low temperatures varies depending on how tolerant a species is to frost damage (Huang et al. 2005; Hajiboland 2012; García-Sánchez et al. 2020; Rahman et al. 2020) It is also possible that *Bo1* or sensitivity to soil boron concentrations may contribute to the signal of the 7B QTL due to a shared abiotic stress tolerance mechanism that is also activated in the presence of LMA.

Another confounding genetic factor that affects LMA expression is the 1B/1R translocation. Originally introduced to hexaploid wheat in the 1950s from a German variety of rye, the 1B/1R translocation harbors several disease resistance genes, as well as genes with the potential to enhance yields and increase environmental adaptability (Ren et al. 2017). The 1B/1R translocation has an additive effect on LMA expression in some LMA-susceptible lines with GA-insensitive, dwarfing genes (Farrell et al. 2013; Mohler et al. 2014). The 1B/1R translocation may be interacting with another part of the genome leading the additive effect to vary across environments and genotypes. Further research is required to understand the influence of the 1B/1R translocation on LMA expression.

Measuring the heritability of the LMA trait has been difficult due to the impact of the environment on expression (Mares and Mrva 2008). It is also the case that LMA expression varies within genotypes and does not consistently present in the same proportion of grains (Derkx and Mares 2020). The link between LMA expression and grain moisture content explains some of this variance as the loss of grain moisture is not uniform across fields, plots, plants, tillers on the same plant, and even grains on the same spike (Mares and Mrva 2008, 2014; Derkx and Mares 2020; Liu et al. 2021). Future research that measures LMA susceptibility by considering grain moisture content over days post-anthesis may lead to more accurate measurements of heritability.

Inaccurate sampling for LMA can affect heritability calculations, but the method of quantifying LMA expression may also influence heritability. Liu et al. (2021) calculated the heritability of LMA at 0.40 when using FN and Phadebas α-amylase enzyme assays to quantify LMA in 242 lines. They noted that Emebiri et al. (2010) calculated a relatively high level of LMA heritability (86.6%) when using an α-amylase ELISA to quantify LMA in an association mapping study of 91 lines. The ELISA test used to quantify LMA is only available in Australia, which has prevented researchers from implementing cohesive methodologies, limiting the ability to draw direct comparisons between studies. In addition, the LMA-ELISA suffers from batch-to-batch variation and generates results that are not reproducible (Neoh et al. 2021). The heritability of LMA is further complicated by LMA being a multi-genic trait with multi-allelic QTL. A study that examined the haplotypes of the genomic regions surrounding the 3B and 7B QTL identified multiple alleles for both loci (McNeil et al. 2009). The 3B QTL had 3 significant alleles, while the 7B QTL had 13. A study characterizing the effect of different alleles at each LMA-associated QTL on LMA expression is yet to be published. It is possible that previously published studies have attempted to compare heritability for LMA in mapping populations which contain different combinations of alleles at the major QTL. Future studies that account for the specific alleles at LMA-associated loci may be able to estimate the heritability of LMA more accurately.

The past 25 years of research on LMA has greatly expanded our understanding of the underlying genetic mechanisms that lead to this phenomenon. As the role of GA sensitivity has become clearer, investigations into the source of LMA resistance should focus on lines that produce low levels of α-amylase even in GA-sensitive cultivars. Additionally, efforts to create near-isogenic lines with different alleles of high-pI α-amylase genes could aid in our understanding of how different alleles and gene copy numbers influence LMA expression and contribute to extreme LMA phenotypes.

## Biochemical mechanisms of LMA

The underlying biochemical mechanisms associated with the induction of LMA are currently unknown. Studies have provided insights into the expression and protein accumulation of α-amylase during this developmental event (Gale et al. 1987; Mrva and Mares 1996c, 1999, 2001a; Joe et al. 2005; Mrva et al. 2006, 2009; Farrell and Kettlewell 2008; Barrero et al. 2013, 2020; Farrell et al. 2013; Kondhare et al. 2013, 2014; Cheng et al. 2014; Mieog et al. 2017; Derkx and Mares 2020). In LMA-susceptible wheat varieties, the relative expression and protein abundance of high pI α-amylase increased during late grain development, at 20 days post-anthesis (dpa) (Barrero et al. 2013). Protein levels continued to rise until 32–35 dpa and remained at maximum levels until harvest ripeness. *TaAMY 1–1* and *TaAMY 1–2* relative expression levels decreased to baseline levels in the majority of LMA-susceptible varieties at 26 dpa (Barrero et al. 2013). Mieog et al. (2017) showed that *TaAMY4* was also expressed during LMA, but TaAMY4 protein levels and activity during this process remain unclear. Since previous studies have not detected high pI α-amylase protein in developing grains prior to 20 dpa or in LMA-inducible varieties that did not receive a temperature stimulus, the α-amylase protein may be a result of new protein synthesis (Mrva et al. 2006; Barrero et al. 2013). Based on these studies, LMA has been defined as a genetic defect that results in the synthesis of high-pI α-amylase enzymes, primarily TaAMY1, during the late stages of grain development. It is well established that high pI α-amylase is synthesized in the aleurone of germinating grains (Cejudo et al. 1995; Appleford and Lenton 1997; Mrva et al. 2006; Cheng et al. 2014) and in isolated aleurone treated with GA (Gubler et al. 1995; Mrva et al. 2006; Barrero et al. 2013). However, the underlying signaling pathways and molecular components involved in the expression of high pI α-amylase during LMA are not well defined.

A gene expression study using constitutive and resistant LMA cultivars provided some clues about the signaling processes and cellular changes associated with LMA (Barrero et al. 2013). In this study, 56 transcripts were significantly more abundant in constitutive LMA cultivars. Some of the transcripts are involved in programmed cell death (PCD), GA response, stress response, and abscisic acid (ABA) signaling (Barrero et al. 2013) and support previous work that implicates these processes in LMA expression (Gale et al. 1987; Mrva and Mares 1996b, 2001b; Mrva et al. 2006; Kondhare et al. 2013, 2014; Derkx and Mares 2020; Derkx et al. 2021). The GA and ABA response during LMA appears to be different from the pathways present in mature aleurone (Barrero et al. 2013; Kondhare et al. 2013, 2014). For example, GA-responsive genes, including *GAMYB*, *(1,3;1,4)-b-glucanase*, *triticain-a,* and *triticain-g*, were not induced by GA in LMA constitutive cultivars during late grain development (Barrero et al. 2013). In addition, the timing and induction of LMA is associated with a change in GA sensitivity, but not ABA sensitivity (Kondhare et al. 2014). Because these studies on LMA have primarily focused on isolated aleurones (Barrero et al. 2013) or half-grains (Kondhare et al. 2012, 2014), these results may be misleading. Future studies focused on gene expression and hormone sensitivity in individual aleurone cells, such as single-cell RNA sequencing, could provide a more accurate picture of the changes in the 2% of cells experiencing LMA (Mrva et al. 2006).

Although LMA appears to only induce the expression and protein synthesis of TaAMY1 and TaAMY4, some of the developmental processes associated with additional hydrolytic enzyme expression and germination are present in the aleurone of LMA-susceptible wheat varieties (Mrva et al. 2006). Mrva et al. (2006) showed that aleurone cells had symptoms of PCD during grain development and at maturity. The PCD symptoms in mature aleurone layers of Spica and Kennedy, two LMA-susceptible varieties, resemble aleurone cells after 48 h of germination. Alternatively, Barrero et al. (2013) reported no visible differences in the aleurone cells of LMA constitutive varieties at 32 dpa. This may be due to the startling low percentage of dead cells in the aleurone of LMA-susceptible varieties or in LMA-resistant varieties after 24 h of germination (Mrva et al. 2006). Germinated grain showed about 2.3% dead cells after 24 h and LMA-susceptible varieties showed about 1.9–2.1% dead cells at maturity. In contrast to LMA, the percentage of dead cells increased from 2 to 100% after 120 h of germination (Mrva et al. 2006).

Another major difference in LMA-susceptible varieties was the location of cells showing symptoms of PCD. In germinating grains, aleurone cells positioned near the scutellum initially showed symptoms of PCD (Mrva et al. 2006). Over time, PCD symptoms spread directionally from the embryo side to the brush side of the grain. In LMA-susceptible varieties, PCD symptoms are present in individual cells or cell patches in random locations throughout the aleurone (Mrva et al. 2006). Previous studies have also shown that LMA initially affected cells near the grain crease in UK varieties (Major 1999; Lunn et al. 2001; Joe et al. 2005). The low percentage of affected cells agrees with the relatively lower levels of α-amylase when mature LMA-affected varieties are compared to sprouted grain over time. The levels of α-amylase in LMA-affected grain correspond to the quantity of α-amylase in germinating grain after 20 h of imbibition (Mrva et al. 2006).

**Fig. 1:**
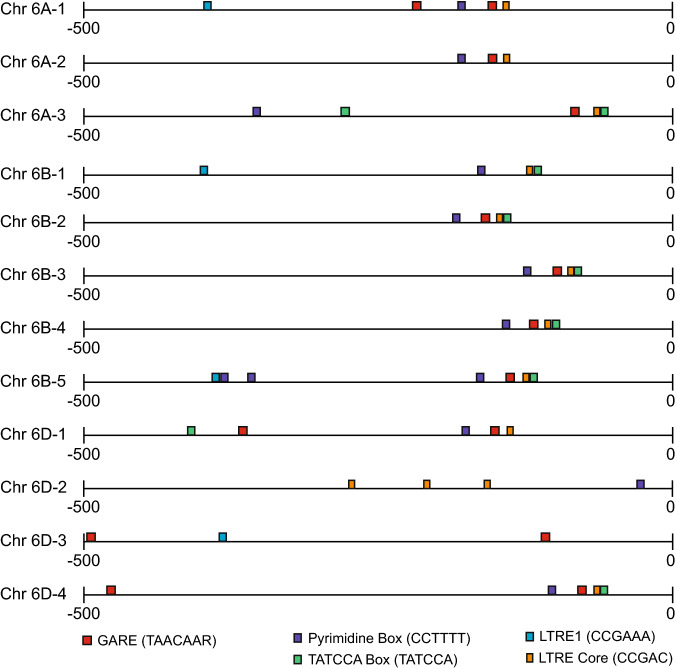
Motifs common to GA-responsive, α-amylase genes and involved in response to low temperature were identified in *TaAMY1* promoter regions. The 500 bp promoter regions of *TaAMY1* genes were analyzed for common, plant-specific promoter motifs. Most *TaAMY1* promoter regions contained motifs involved in α-amylase gene expression regulation. In addition, regions that could be involved in the cold-induced expression of *AMY1* were also identified. This figure was made using Adobe Illustrator. (Skriver et al. 1991; Gubler and Jacobsen 1992; Baker et al. 1994; Rogers et al. 1994; Jiang et al. 1996; Dunn et al. 1998; Higo et al. 1999)

Because some characteristics of LMA appear to be different from the process of germination and from the GA-induced production of α-amylase in mature aleurone, the regulation of high pI α-amylase gene expression may also be distinct. During germination and in mature barley aleurone, GA signaling is initiated by GA binding to and activating a receptor (Lovegrove and Hooley 2000). The activated receptor may interact with and initiate a signaling cascade that includes a heterotrimeric G-protein complex (Jones et al. 1998), Ca^2+^ (Bush 1996), cyclic GMP (cGMP), kinases, and phosphatases (Bethke et al. 1997; Ritchie and Gilroy 1998; Lovegrove and Hooley 2000). This pathway is regulated by the transcription factors, SLN1 and SLR1 (Ikeda et al. 2001; Chandler et al. 2002). Downstream of the initiation of this pathway, the transcription factor, GAMYB, transactivates the expression of high pI α-amylase (Gubler et al. 1995, 2002) Studies have shown that GAMYB is necessary and sufficient for the expression of *TaAMY1* (Gubler et al. 1995; Zentella et al. 2002). GAMYB binds to a region in the *AMY1* promoter termed the GA Response Element (GARE) (Gubler et al. 1995). To determine if wheat *AMY1* promoters include similar regulatory regions, we first identified the chromosome location of *Triticum aestivum α-amylase1 (TaAMY1)* genes using GenBank and accession numbers from Mieog et al. (2017). The 500-base pair (bp) region upstream of the transcriptional start site of *TaAMY1* genes was used to scan for plant cis-acting regulatory DNA elements using the PLACE Database (Higo et al. 1999). We located at least one GARE in most *TaAMY1* promoter regions (Fig. 1). In addition to GAREs, the pyrimidine and TATCCAC box were also located in the promoter region of several *TaAMY1* gene promoters. Together, the GARE, pyrimidine, and TATCCAC box make up the GA response complex (GARC) in promoters of plant α-amylase genes and play important roles in GA-regulated expression (Skriver et al. 1991; Gubler and Jacobsen 1992; Rogers et al. 1994). Promoter elements that could be involved in the cold-induced expression of *AMY1* were also identified and labeled as low-temperature response elements (LTRE) (Fig. 1) (Baker et al. 1994; Jiang et al. 1996; Dunn et al. 1998). Interestingly, GAMYB is also detectable in barley aleurone that have not been treated with GA and without the accumulation of α-amylase activity (Gubler et al. 2002). If *GAMYB* is expressed and maintained in aleurone cells in the absence of GA, this may explain how high pI α-amylase is expressed during LMA when there is no detectable change in *GAMYB* expression.

In contrast to GA activation, the expression of *TaAMY1* is suppressed by ABA signaling components (Gómez-Cadenas et al. 1999, 2001; Gubler et al. 2002). The protein kinase, PKABA1, suppresses *TaAMY1* expression by downregulating the transcription of *GAMYB* (Gómez-Cadenas et al. 2001). Another molecular component involved in this complex interplay is the DELLA protein, SLN1 (Gubler et al. 2002). In barley, SLN1 repressed the expression of *GAMYB*. However, SLN1 protein levels decline in response to GA, and this leads to an increase in the expression of *GAMYB* and *TaAMY1* (Gubler et al. 2002). The complex network of GA and ABA signaling components and mechanisms are likely involved in the activation of *TaAMY1* and/or *TaAMY4* expression and protein accumulation during LMA. Hypothetically, disruption in the expression or regulation of these molecular components could lead to changes in accumulation of the TaAMY1 and/or TaAMY4 protein that are characteristic of LMA.

Notably, a recent study identified the gene, *LMA1*, as a genetic cause of variation in LMA phenotypes (Derkx et al. 2021). From a biochemical perspective, this gene encodes a protein with homology to an enzyme involved in the first step of GA biosynthesis (Wu et al. 2012; Toyomasu et al. 2015). In LMA resistant varieties, the LMA1 protein sequence was changed or there was an introduction of a premature stop codon (Derkx et al. 2021). A functional *LMA1* gene led to LMA susceptibility in some varieties and curiously, a functional allele of this gene was shown to be the more common, wild-type-like condition. If this is the case, LMA may not be a genetic defect and may not only be present in a few varieties (Mares and Mrva 2014; Barrero et al. 2020). This shift in understanding leads to new questions about LMA and the prevalence of this condition in cultivated wheat varieties. For example, what gene or mechanism(s) are missing in varieties where a functional LMA1 protein is present, but it does not lead to LMA susceptibility? Furthermore, as mentioned above, previous studies have suggested that GA signaling may be involved in LMA expression (Mrva and Mares 1996b; Farrell et al. 2013; Derkx and Mares 2020). Future research focused on the functional role of LMA1 in GA biosynthesis during grain development and germination will provide further insights into why a functional *LMA1* allele can lead to LMA susceptibility.

## Physiological mechanisms of LMA

To understand and fully describe the internal chemical and physical processes that define the underlying physiological mechanisms of LMA, it is important to describe the roles that hormone signaling networks play in regulating wheat grain germination. At the time of dispersal, seeds can be dormant and fail to germinate immediately after transport. In this way, seed dormancy is an evolutionary adaptation that prevents precocious germination on the mother plant or in unfavorable environments where soil moisture or nutrients are limited (Bewley et al. 2012; Shu et al. 2015, 2016; Penfield 2017). During embryo development, dormancy is regulated by the plant hormones, ABA and auxin, and decreases over time in response to environmental conditions including light, temperature, and moisture levels (Finkelstein et al. 2002, 2008; Bentsink and Koornneef 2008; Graeber et al. 2012; Liu et al. 2013; Shu et al. 2015; Shuai et al. 2016; Matilla 2020). As dormancy is lost, germination capacity increases, and the transition from a dormant to non-dormant state occurs. This developmental shift is accompanied by a decrease in ABA levels and signaling and an increase in GA accumulation and signaling (Koornneef et al. 1982; Karssen and Laçka 1986; Olszewski et al. 2002; Kushiro et al. 2004; Okamoto et al. 2006; Tuan et al. 2018).

Once dormancy is lost, germination begins with water uptake and ends with radicle emergence (Bewley et al. 2012). GA-induced signaling triggers embryonic growth and the release of hydrolytic enzymes that help to weaken the seed coat and break down storage reserves that provide energy for embryogenesis and seedling establishment (Peng and Harberd 2002; Gao and Chu 2020). In cereals, GA signaling stimulates the expression of α-amylase enzymes in the aleurone that are secreted and subsequently degrade starch in the endosperm (Fincher 1989; Jones and Jacobsen 1991; Gubler et al. 1995). As mentioned previously, elevated α-amylase expression and protein accumulation, resulting from diminished seed dormancy or occurring outside of the process of germination, are the primary indicators of PHS and LMA (Mares and Mrva 2014). Although both conditions result in the expression of relatively high levels of α-amylase, the level of expression, timing of induction, and location of enzyme activity differ. Studies in wheat and barley have demonstrated that PHS tolerance and seed dormancy are associated with relatively higher ABA sensitivity, while PHS susceptibility is associated with relatively higher GA sensitivity (Walker-Simmons 1987; Morris et al. 1989; Barrero et al. 2009; Schramm et al. 2010; Tuttle et al. 2015). In addition, during PHS, α-amylase levels are highest near the embryo end of the grain, a trend that is consistent with germination (Mares et al. 1994; Mares and Mrva 2014). In other words, PHS mirrors the process of germination, it is just happening prior to harvest. Unlike PHS, LMA results in the expression and protein accumulation of α-amylase throughout the aleurone layer (Mrva et al. 2006; Mares and Mrva 2008). Although the phenotypic manifestation of LMA is distinct, studies have demonstrated that components of GA signaling may also be involved in LMA (Kondhare et al. 2012, 2013, 2014; Barrero et al. 2013).

Historically, the role of GA signaling during seed development in cereals has been controversial. This is in part because bioactive GAs are difficult to measure, and the expression of GA biosynthesis genes prior to germination is typically low (Jacobsen et al. 2002; Ogawa et al. 2003; Yamauchi et al. 2004; Ueguchi-Tanaka et al. 2005; Barrero et al. 2009). Additionally, studies evaluating GA responses in the barley DELLA *Slender 1* (*Sln1*) mutants indicate that GA hormone may not be necessary for stimulating responses downstream of SLN1 (Chandler et al. 2002; Chen et al. 2010). Research focused on the regulation of dormancy in Arabidopsis has shown that the GA receptor, GID1, is present in seeds even in the absence of GA biosynthesis (Hauvermale et al. 2015; Hauvermale and Steber 2020). Collectively, these observations suggests that for seeds to complete the successful transition between germination and seedling establishment, not only do components of GA signaling need to be in place before germination is initiated, but in some instances DELLA regulation may occur through GA-independent and/or non-proteolytic mechanisms (Chandler et al. 2002; Chen et al. 2010; Hauvermale et al. 2012). In a similar way, GA signaling components may also be required during the developmental transition between embryo development and seed maturation, the time when LMA events are likely to occur. Furthermore, enhanced GA signaling during LMA could also occur through changes in GID1 receptor accumulation or binding. Functional studies have demonstrated that some GID1 isoforms have evolved to bind DELLA proteins and initiate a relatively low level of GA signaling in the absence of GA (Hauvermale et al. 2015; Nelson and Steber 2016; Hauvermale and Steber 2020). While studies in wheat suggest that GA signaling most often occurs through GA binding to GID1, SNP analysis of the D genome donor, *Aegilops tauschii* Coss., has identified modifications in *GID1* gene sequences that may lead to altered GID1 function and provides another route by which enhanced or altered GA signaling may contribute to LMA (Bazhenov et al. 2020). It has also been suggested that cereal aleurone cells may have a GA-perception system that is different than the GID1-DELLA-mediated GA-perception system (Ueguchi-Tanaka et al. 2007). This hypothesis is supported by studies showing that aleurone cells cannot produce bioactive GA, but can only perceive GA that has been produced by the embryo and transported to the aleurone (Kaneko et al. 2003). In addition, Gilroy and Jones showed that α-amylase expression was not induced when GA was injected into barley aleurone cells (Gilroy and Jones 1994). Finally, there is also evidence that heterotrimeric G-proteins play a role in GA signaling in the aleurone (Ashikari et al. 1999; Lovegrove and Hooley 2000; Ueguchi-Tanaka et al. 2000). Collectively, these experiments suggest that the site of GA perception is outside of the plasma membrane and that the GA-receptor in these cells may be located in the plasma membrane. Although, in *gid1* aleurone cells, GA-induced α-amylase expression is relatively low (Ueguchi-Tanaka et al. 2005). A unique GA perception system in the aleurone could provide an additional route by which changes in GA signaling or GA sensitivity could lead to LMA during wheat grain development.

In some LMA-susceptible varieties, a cold shock is required to initiate α-amylase expression and protein accumulation (Mrva and Mares 2001b; Tan et al. 2010). In addition, a cold shock can enhance the expression of high pI α-amylase in LMA constitutive varieties (Derkx and Mares 2020; Derkx et al. 2021). Similarly, cold treatment during seed imbibition functions to synchronize seed germination and increases GA levels and sensitivity to GA (Yamauchi et al. 2004). The purpose of enhanced GA signaling and sensitivity resulting from cold imbibition may be to jump start the germination process to ensure seedling establishment (Hauvermale and Steber 2020). Therefore, it is interesting to consider that cold increases the amount of α-amylase accumulation in LMA-susceptible and LMA-constitutive cultivars (Mrva and Mares 2001b; Derkx and Mares 2020; Derkx et al. 2021). This observation suggests that GA accumulation and signaling may be an important component of LMA regulation that is enhanced by cold (Derkx et al. 2021). An alternative hypothesis is that LMA-associated physiological changes that are induced by a cold snap, or other environmental stressors, may activate sensing machinery and signaling cascades that function to protect the developing embryo. Many studies have demonstrated that plants show specific changes in gene expression, metabolism, and physiology in response to environmental stressors and that these changes occur through cell specific sensing machinery (Zhu 2016). For example, the phytochrome photoreceptors and downstream signaling pathway have been implicated in thermosensing and facilitating adaptive responses (Jung et al. 2016; Legris et al. 2019). After abiotic stress cues are perceived, there is substantial overlap in the signaling molecules that induce the cellular response (Lamers et al. 2020). This suggests that plants and seeds may sense specific stresses during development, and that each cell or cell type may have specific sensing machinery, but common molecular components are used to activate the downstream cellular changes. In relation to LMA, there may be cell specific stress sensors and varying sensitivities to stress or stress-induced signals within the aleurone (Ritchie et al. 1999). This may explain the stochastic expression and protein accumulation of α-amylase in random cells or cell patches throughout the aleurone during LMA.

Another hallmark of cereal grain germination is the PCD of the aleurone after hydrolytic enzyme synthesis and secretion (Kuo et al. 1996; Bethke et al. 1999; Fath et al. 2000). In contrast to this tightly regulated developmental process, the cellular changes characteristic of cell death and associated with LMA are localized to individual or small groups of aleurone cells and do not appear to affect the overall integrity of the aleurone layer (Mrva et al. 2006). Thus, the possibility exists that LMA may be part of an apoptosis-like process. Although apoptosis has not been well characterized in plants, it is well described and highly conserved across living organisms and is tightly regulated to function in specific cells at specific times (Hengartner 2000; Dickman et al. 2017). Therefore, future work will need to evaluate if the cell death occurring in the aleurone during LMA occurs as a consequence of PCD or apoptosis.

Overall, the underlying physiological processes of LMA are distinct from what is seen during cereal seed germination. Some of the same plant metabolites, signaling processes, and molecular components may be involved in facilitating this developmental process. For example, there is plenty of evidence supporting a role for GA and GA-signaling components in the expression of LMA. Future studies focused on identifying LMA-specific metabolites, signaling mechanisms, and molecular components will provide insight into the chemical and physical aspects of this process. In addition, these studies will increase our fundamental understanding of stress sensing and response during seed development.

## Effects of LMA on end-use quality

Much less is known about the effects of LMA on wheat quality compared to those of PHS. The falling number test was originally designed for PHS-affected grain. As such, historic correlations between FN and wheat quality are fairly limited to PHS only. More recently, LMA has been examined more intensively and likely affects wheat quality differently than does PHS (Kiszonas et al. 2018; Newberry et al. 2018). The association between LMA and wheat quality has been studied with hard wheat, primarily used for making bread (Newberry et al. 2018). One limitation of the Newberry et al. study, however, was that the range of FN was limited to 193–294 s, all below typical contract specifications for FN values of export wheat, and also below the domestic use baking company specifications (2018). In bread, there has been no difference across a FN range of 193–294 s for loaf volume or crumb structure (Newberry et al. 2018). In a study with overexpressed *TaAMY3*, bread volume increased, particularly with addition of a baking improver (Ral et al. 2016). Similarly, noodle firmness was unaffected by high α-amylase levels in grain that had overexpressed *TaAMY3*, although cooking loss was substantially greater for lines with higher levels of α-amylase (Ral et al. 2018). As such, with bread and noodles, there are mixed results for LMA-affected grain. Bread appears to be unaffected, or improved by LMA-affected grain, though noodles show a decrease in quality for the cooking loss parameter.

Soft wheat, used for cookies, cakes, and pastries, is typically strongly influenced by low FN typically attributed to PHS (Finney et al. 1981; Edwards et al. 1989; Kiszonas et al. 2018). Japanese sponge cakes, in particular, are dramatically affected by PHS-affected grain, producing very small, dense, highly unacceptable cakes because of small size and very firm crumb (Finney et al. 1981; Kiszonas et al. 2018). An unusual result reported in Kiszonas et al. (2018) was a wide range of FN values, with some very low FN samples having seemingly acceptable cake volume and quality. The hypothesis was that these samples may have had low FN due to LMA instead of PHS, though future studies will need to focus on LMA-verified grain.

The challenge of obtaining verified-LMA affected samples in a sufficient quantity for milling and baking results in few studies about LMA-affected wheat quality. Newberry et al. (2018) acknowledged this challenge but did obtain samples with FN from 193 to 294 s and found no definitive correlations between FN and bread loaf volume or other bread quality traits. The stated hypotheses about the lessened effect of LMA-affected grain compared to PHS-affected grain are largely based on anecdotal data and observations over long study of cake quality. A critical step moving forward will be to obtain LMA-verified grain at varied FN in a sufficient quantity to perform extensive milling and baking tests. These types of experiments would greatly expand the knowledge and understanding of how LMA-affected grain impacts end-use quality. Additionally, the findings that LMA-affected grain had a positive impact on bread may allow for some grain handling and supply chain changes that allow LMA-verified grain to be supplied directly to bakeries focusing on bread. If LMA-affected grain could be redirected to these specialty supply chains, its value could increase as opposed to the traditional economic penalty of having low FN grain.

## Concluding remarks

In the last decade, studies focused on the underlying genetic, biochemical, and physiological mechanisms of LMA have become increasingly important due to large financial losses resulting from low FN in the absence of sprouting (Kingwell and Carter 2014; Mares and Mrva 2014; Steber 2017; Bettge 2018). Unfortunately, the complexity and variability of this trait make it a challenging topic to study. Studies focused on the genetics of LMA have identified several QTL that contribute to LMA expression. In addition, these studies have concluded that LMA is a multi-genic and multi-allelic trait this is heavily influenced by the environment. As more LMA associated QTLs are linked to functional genes, our understanding of the underlying mechanism(s) that lead to LMA will increase and molecular markers of LMA susceptibility or resistance can be developed.

LMA is defined as the expression and protein accumulation of high pI α-amylase during grain development. Studies focused on identifying the underlying biochemical and physiological mechanisms that lead LMA expression have not yet identified any specific signal(s), signaling mechanisms, or cellular environments. Single cell technologies (Rich-Griffin et al. 2020; Thibivilliers and Libault 2021), a recently expanded wheat genome release (Walkowiak et al. 2020; Zhu et al. 2021), and improved plant transformation methods (Demirer et al. 2019) will help wheat researchers to continue unraveling the complex mechanisms that lead to LMA in wheat. In addition, we will gain a better understanding of how to balance the need for rapid and effective germination versus controlling the damaging activity of α-amylase prior to harvest. Changing climates with unpredictable temperature fluctuations could lead to LMA occurring more frequently and as a result have created a sense of urgency for scientists working on understanding and solving this wheat quality issue.

To date, most literature classifies LMA as a genetic defect, which means that LMA results in a disruption of normal physiological processes during grain development. While the expression of high levels of α-amylase may negatively impact end-use quality in some instances, there is little evidence to suggest that LMA has a negative impact on seed development, yield, viability, germination potential, or dispersal. Additionally, the discovery that *LMA-1* encodes a wild-type copy of a CPS gene suggests LMA may be a normal part of seed development (Derkx et al. 2021). In contrast, the *shruken2* mutation in maize, which causes premature PCD-associated aleurone death during development, has a negative impact on germination potential and seed viability (Young et al. 1997). Therefore, future research will need to carefully explore the environmental, genetic, and biochemical processes that lead to LMA, and to parse apart its fundamental roles both, negative and positive, in cereal grain development.

Finally, although there is a strong interest in understanding the underlying chemical and physical processes that lead to LMA expression, it will also be important to determine if LMA has a negative effect on end-use quality. Previous studies focused on the end-use quality effects of LMA-affected grain have provided encouraging but incomplete results (Kiszonas et al. 2018; Newberry et al. 2018). A comprehensive understanding of LMA from the underlying molecular aspects to the end-use quality effects will greatly benefit the global wheat industry and those whose livelihoods depend upon it.

### *Author contributions statement*

AEC conceived and proposed the review topic. EJM, AMK, ALH, DRS and AEC wrote portions of the review. All authors read and approved the manuscript.

## Data Availability

The datasets generated during and/or analyzed during the current study are available from the corresponding author on reasonable request.

## References

[CR1] Appleford NEJ, Lenton JR (1997). Hormonal regulation of α-amylase gene expression in germinating wheat (*Triticum aestivum*) grains. Physiol Plant.

[CR2] Ashikari M, Wu J, Yano M (1999). Rice gibberellin-insensitive dwarf mutant gene dwarf 1 encodes the α-subunit of GTP-binding protein. PNAS.

[CR3] Baker SS, Wilhelm KS, Thomashow MF (1994). The 5’-region of *Arabidopsis thaliana* cor15a has cis-acting elements that confer cold-, drought- and ABA-regulated gene expression. Plant Mol Biol.

[CR4] Barrero JM, Talbot MJ, White RG (2009). Anatomical and transcriptomic studies of the coleorhiza reveal the importance of this tissue in regulating dormancy in barley. Plant Physiol.

[CR5] Barrero JM, Mrva K, Talbot MJ (2013). Genetic, hormonal, and physiological analysis of late maturity α-amylase in wheat. Plant Physiol.

[CR6] Barrero JM, Porfirio L, Hughes T (2020). Evaluation of the impact of heat on wheat dormancy, late maturity α-amylase and grain size under controlled conditions in diverse germplasm. Sci Rep.

[CR7] Bazhenov MS, Chernook AG, Goncharov NP (2020). The allelic diversity of the gibberellin signaling pathway genes in *Aegilops tauschii* coss. Plants (basel).

[CR8] Bell R (2017). Mobility and distribution of boron in plants and effects on reproductive growth and yield. J Boron.

[CR9] Bentsink L, Koornneef M (2008). Seed dormancy and germination. Arabidopsis Book.

[CR10] Bethke PC, Schuurink R, Jones RL (1997). Hormonal signalling in cereal aleurone. J Exp Bot.

[CR11] Bethke PC, Lonsdale JE, Fath A, Jones RL (1999). Hormonally regulated programmed cell death in barley aleurone cells. Plant Cell.

[CR12] Bettge A (2018). Low falling numbers in the pacific northwest wheat growing region: preharvest sprouting, late maturity amylase, falling number instrument, or low protein?. Cereal Foods World.

[CR13] Bevan JG (2020) Comparative QTL Mapping of Hagberg Falling Number, Pre-Harvest Sprouting, and Late Maturity Alpha-amylase in UI Platinum by SY Capstone Derived Population - ProQuest. Master of Science, University of Idaho

[CR14] Bewley JD, Bradford K, Hilhorst H, Nonogaki H (2012). Seeds: physiology of development, germination, and dormancy.

[CR15] Bingham J, Whitmore ET (1966). Varietal differences in wheat in resistance to germination in the ear and α-amylase content of the grain. J Agric Sci.

[CR16] Börner A, Nagel M, Agacka-Mołdoch M (2018). QTL analysis of falling number and seed longevity in wheat (*Triticum aestivum* L.). J Appl Genetics.

[CR17] Brdar-Jokanović M (2020). Boron toxicity and deficiency in agricultural plants. Int J Mol Sci.

[CR18] Bush DS (1996). Effects of gibberellic acid and environmental factors on cytosolic calcium in wheat aleurone cells. Planta.

[CR19] Cejudo FJ, Cubo MT, Baulcombe DC (1995). *Amyl* expression during wheat seed germination. Plant Sci.

[CR20] Chandler PM, Marion-Poll A, Ellis M, Gubler F (2002). Mutants at the *Slender1* Locus of Barley cv Himalaya. Molecular and physiological characterization. Plant Physiol.

[CR21] Chen K, Tian S, Yandell BS (2010). Loss-of-function of DELLA protein SLN1 activates GA signaling in barley aleurone. Acta Physiol Plant.

[CR22] Cheng C-R, Oldach K, Mrva K, Mares D (2014). Analysis of high pI α-amy-1 gene family members expressed in late maturity α-amylase in wheat (*Triticum aestivum* L.). Mol Breed.

[CR23] Demirer GS, Zhang H, Matos JL (2019). High aspect ratio nanomaterials enable delivery of functional genetic material without DNA integration in mature plants. Nat Nanotechnol.

[CR24] Derkx AP, Mares DJ (2020). Late-maturity α-amylase expression in wheat is influenced by genotype, temperature and stage of grain development. Planta.

[CR25] Derkx A, Baumann U, Cheong J (2021). A major locus on wheat chromosome 7B associated with late-maturity α-amylase encodes a putative ent-copalyl diphosphate synthase. Front Plant Sci.

[CR26] Dickman M, Williams B, Li Y (2017). Reassessing apoptosis in plants. Nat Plants.

[CR27] Dobrotvorskiy D, Dobrotvorskaya T, Martynov S (2021) GRIS: Genetic Resources Information System for Wheat and Triticale. http://wheatpedigree.net

[CR28] Dunn MA, White AJ, Vural S, Hughes MA (1998). Identification of promoter elements in a low-temperature-responsive gene (*blt4.9*) from barley (*Hordeum vulgare* L.). Plant Mol Biol.

[CR29] Edwards RA, Ross AS, Mares DJ (1989). Enzymes from rain-damaged and laboratory-germinated wheat I. Effects on product quality. J Cereal Sci.

[CR30] Emebiri LC, Oliver JR, Mrva K, Mares D (2010). Association mapping of late maturity α-amylase (LMA) activity in a collection of synthetic hexaploid wheat. Mol Breed.

[CR31] Farrell AD, Kettlewell PS (2008). The effect of temperature shock and grain morphology on alpha-amylase in developing wheat grain. Ann Bot.

[CR32] Farrell AD, Kettlewell PS, Simmonds J (2013). Control of late maturity alpha-amylase in wheat by the dwarfing gene Rht-D1b and genes on the 1B/1R translocation. Mol Breed.

[CR33] Fath A, Bethke P, Lonsdale J (2000). Programmed cell death in cereal aleurone. Plant Mol Biol.

[CR34] Fincher GB (1989). Molecular and cellular biology associated with endosperm mobilization in germinating cereal grains. Annu Rev Plant Physiol Plant Mol Biol.

[CR35] Finkelstein RR, Gampala SSL, Rock CD (2002). Abscisic acid signaling in seeds and seedlings. Plant Cell.

[CR36] Finkelstein R, Reeves W, Ariizumi T, Steber C (2008). Molecular aspects of seed dormancy. Annu Rev Plant Biol.

[CR37] Finney KF, Natsuaki O, Bolte LC (1981). Alpha-amylase in field-sprouted wheats: its distribution and effect on Japanese-type sponge cake and related physical and chemical tests. Cereal Chem.

[CR38] Ford BA, Foo E, Sharwood R (2018). Rht18 semidwarfism in wheat is due to increased GA 2-oxidaseA9 expression and reduced GA content. Plant Physiol.

[CR39] Gale MD, Marshall GA (1975). The nature and genetic control of gibberellin insensitivity in dwarf wheat grain. Heredity.

[CR40] Gale MD, Salter AM, Lenton JR (1987) The Induction of Germination Alpha-Amylase During Wheat Grain Development in Unfavorable Weather Conditions. Fourth International Symposium on Pre-Harvest Sprouting in Cereals, Boulder, Co, USA 273–282

[CR41] Gao S, Chu C (2020). Gibberellin metabolism and signaling: targets for improving agronomic performance of crops. Plant Cell Physiol.

[CR42] García-Sánchez F, Simón-Grao S, Martínez-Nicolás JJ (2020). Multiple stresses occurring with boron toxicity and deficiency in plants. J Hazard Mater.

[CR43] Gilroy S, Jones RL (1994). Perception of gibberellin and abscisic acid at the external face of the plasma membrane of barley (*Hordeum vulgare* L.) aleurone protoplasts. Plant Physiol.

[CR44] Gómez-Cadenas A, Verhey SD, Holappa LD (1999). An abscisic acid-induced protein kinase, PKABA1, mediates abscisic acid-suppressed gene expression in barley aleurone layers. Proc Natl Acad Sci USA.

[CR45] Gómez-Cadenas A, Zentella R, Walker-Simmons MK, Ho TH (2001). Gibberellin/abscisic acid antagonism in barley aleurone cells: site of action of the protein kinase PKABA1 in relation to gibberellin signaling molecules. Plant Cell.

[CR46] Graeber K, Nakabayashi K, Miatton E (2012). Molecular mechanisms of seed dormancy. Plant Cell Environ.

[CR47] Gubler F, Jacobsen JV (1992). Gibberellin-responsive elements in the promoter of a barley high-pI alpha-amylase gene. Plant Cell.

[CR48] Gubler F, Kalla R, Roberts JK, Jacobsen JV (1995). Gibberellin-regulated expression of a Myb gene in barley aleurone cells: evidence for Myb transactivation of a high-pi alpha-amylase gene promoter. Plant Cell.

[CR49] Gubler F, Chandler PM, White RG (2002). Gibberellin signaling in barley aleurone cells. Control of *SLN1* and *GAMYB* expression. Plant Physiol.

[CR50] Hagberg S (1960). A rapid method for determining alpha-amylase activity. Cereal Chem.

[CR51] Hagberg S (1961). Note on a simplified rapid method for determining alpha-amylase activity. Cereal Chem.

[CR52] Hajiboland R (2012). Effect of micronutrient deficiencies on plants stress responses. Abiotic Stress Responses Plants.

[CR53] Hauvermale AL, Steber CM (2020). GA signaling is essential for the embryo-to-seedling transition during arabidopsis seed germination, a ghost story. Plant Signal Behav.

[CR54] Hauvermale AL, Ariizumi T, Steber CM (2012). Gibberellin signaling: a theme and variations on DELLA repression. Plant Physiol.

[CR55] Hauvermale AL, Tuttle KM, Takebayashi Y (2015). Loss of *Arabidopsis thaliana* seed dormancy is associated with increased accumulation of the GID1 GA hormone receptors. Plant Cell Physiol.

[CR56] Hengartner MO (2000). The biochemistry of apoptosis. Nature.

[CR57] Higo K, Ugawa Y, Iwamoto M, Korenaga T (1999). Plant cis-acting regulatory DNA elements (PLACE) database: 1999. Nucleic Acids Res.

[CR58] Huang L, Ye Z, Bell RW, Dell B (2005). Boron nutrition and chilling tolerance of warm climate crop species. Ann Bot.

[CR59] Ikeda A, Ueguchi-Tanaka M, Sonoda Y (2001). Slender rice, a constitutive gibberellin response mutant, is caused by a null mutation of the *SLR1* gene, an ortholog of the height-regulating gene *GAI*/*RGA*/*RHT*/*D8*. Plant Cell.

[CR60] Jacobsen JV, Pearce DW, Poole AT (2002). Abscisic acid, phaseic acid and gibberellin contents associated with dormancy and germination in barley. Physiol Plant.

[CR61] Jiang C, Iu B, Singh J (1996). Requirement of a CCGAC cis-acting element for cold induction of the *BN115* gene from winter *Brassica napus*. Plant Mol Biol.

[CR62] Joe AFTW, Summers RW, Lunn GD (2005). Pre-maturity α-amylase and incipient sprouting in UK winter wheat, with special reference to the variety rialto. Euphytica.

[CR63] Jones RL, Jacobsen JV (1991). Regulation of synthesis and transport of secreted proteins in cereal aleurone. Int Rev Cytol.

[CR64] Jones HD, Smith SJ, Desikan R (1998). Heterotrimeric G proteins are implicated in gibberellin induction of a-amylase gene expression in wild oat aleurone. Plant Cell.

[CR65] Jung J-H, Domijan M, Klose C (2016). Phytochromes function as thermosensors in arabidopsis. Science.

[CR66] Kaneko M, Itoh H, Inukai Y (2003). Where do gibberellin biosynthesis and gibberellin signaling occur in rice plants? Expression patterns of GA-related genes in rice. Plant J.

[CR67] Karssen CM, Laçka E, Bopp M (1986). A revision of the hormone balance theory of seed dormancy: studies on gibberellin and/or abscisic acid-deficient mutants of *Arabidopsis thaliana*. Plant growth substances 1985.

[CR68] Kingwell R, Carter C (2014). Economic issues surrounding wheat quality assurance: the case of late maturing alpha-amylase policy in Australia. Austral Agribus Rev.

[CR69] Kiszonas AM, Engle DA, Pierantoni LA, Morris C (2018). Relationships between falling number, α-amylase activity, milling, cookie, and sponge cake quality of soft white wheat. Cereal Chem.

[CR70] Kondhare KR, Kettlewell PS, Farrell AD (2012). Effects of exogenous abscisic acid and gibberellic acid on pre-maturity α-amylase formation in wheat grains. Euphytica.

[CR71] Kondhare KR, Kettlewell PS, Farrell AD (2013). The role of sensitivity to abscisic acid and gibberellin in pre-maturity α-amylase formation in wheat grains. J Cereal Sci.

[CR72] Kondhare KR, Hedden P, Kettlewell PS (2014). Use of the hormone-biosynthesis inhibitors fluridone and paclobutrazol to determine the effects of altered abscisic acid and gibberellin levels on pre-maturity α-amylase formation in wheat grains. J Cereal Sci.

[CR73] Koornneef M, Jorna ML, Brinkhorst-van der Swan DL, Karssen CM (1982). The isolation of abscisic acid (ABA) deficient mutants by selection of induced revertants in non-germinating gibberellin sensitive lines of *Arabidopsis thaliana* (L.) heynh. Theor Appl Genet.

[CR74] Kuo A, Cappelluti S, Cervantes-Cervantes M (1996). Okadaic acid, a protein phosphatase inhibitor, blocks calcium changes, gene expression, and cell death induced by gibberellin in wheat aleurone cells. Plant Cell.

[CR75] Kushiro T, Okamoto M, Nakabayashi K (2004). The arabidopsis cytochrome P450 CYP707A encodes ABA 8’-hydroxylases: key enzymes in ABA catabolism. EMBO J.

[CR76] Lamers J, van der Meer T, Testerink C (2020). How plants sense and respond to stressful environments. Plant Physiol.

[CR77] Legris M, Ince YÇ, Fankhauser C (2019). Molecular mechanisms underlying phytochrome-controlled morphogenesis in plants. Nat Commun.

[CR78] Liu X, Zhang H, Zhao Y (2013). Auxin controls seed dormancy through stimulation of abscisic acid signaling by inducing ARF-mediated ABI3 activation in arabidopsis. Proc Natl Acad Sci USA.

[CR79] Liu C, Parveen RS, Revolinski SR (2021). The genetics of late maturity alpha-amylase (LMA) in North American spring wheat (Triticum aestivum L.). Seed Sci Res.

[CR80] Lovegrove A, Hooley R (2000). Gibberellin and abscisic acid signaling in aleurone. Trends Plant Sci.

[CR81] Lunn MBJ, Kettlewell PS, Scott RK (2001). Mechanisms leading to excess alpha -amylase activity in wheat (*Triticum aestivum*, L) grain in the U.K. J Cereal Sci.

[CR82] Major BJ (1999) Environmental Factors Affecting Pre-Maturity *Alpha*-Amylase Activity in Winter Wheat (*Triticum aestivum*). Phd, The Open University

[CR83] Mares DJ (1993). Pre-harvest sprouting in wheat. I. Influence of cultivar, rainfall and temperature during grain ripening. Aust J Agric Res.

[CR84] Mares D, Mrva K (2008). Late-maturity α-amylase: low falling number in wheat in the absence of preharvest sprouting. J Cereal Sci.

[CR85] Mares DJ, Mrva K (2014). Wheat grain preharvest sprouting and late maturity alpha-amylase. Planta.

[CR86] Mares DJ, Mrva K, Panozzo JF (1994). Characterization of the high α -amylase levels in grain of the wheat cultivar BD 159. Aust J Agric Res.

[CR87] Martinez SA, Godoy J, Huang M (2018). Genome-wide association mapping for tolerance to preharvest sprouting and low falling numbers in wheat. Front Plant Sci.

[CR88] Matilla AJ (2020). Auxin: hormonal signal required for seed development and dormancy. Plants (basel).

[CR89] McNeil MD, Diepeveen D, Wilson R (2009). Haplotype analyses in wheat for complex traits: tracking the chromosome 3B and 7B regions associated with late maturity alpha-amylase (LMA) in breeding programs. Crop Pasture Sci.

[CR90] Mieog JC, Janeček Š, Ral J-P (2017). New insight in cereal starch degradation: identification and structural characterization of four α-amylases in bread wheat. Amylase.

[CR91] Mohan A, Grant NP, Schillinger WF, Gill KS (2021). Characterizing reduced height wheat mutants for traits affecting abiotic stress and photosynthesis during seedling growth. Physiol Plant.

[CR92] Mohler V, Albrecht T, Mrva K (2014). Genetic analysis of falling number in three bi-parental common winter wheat populations. Plant Breed.

[CR93] Morris CF, Moffatt JM, Sears RG, Paulsen GM (1989). Seed dormancy and responses of caryopses, embryos, and calli to abscisic acid in wheat. Plant Physiol.

[CR94] Mrva K, Mares DJ (1996a) Control of Later Maturity α-Amylase Synthesis Compared to Enzyme Synthesis During Germination. In: The Seventh International Symposium on Pre-Harvest Sprouting in Cereals. Center for Academic Societies Japan, pp 419–426

[CR95] Mrva K, Mares DJ (1996). Expression of late maturity alpha-amylase in wheat containing gibberellic acid insensitivity genes. Euphytica.

[CR96] Mrva K, Mares DJ (1996). Inheritance of late maturity alpha-amylase in wheat. Euphytica.

[CR97] Mrva K, Mares DJ (1999). Regulation of high pI α-amylase synthesis in wheat aleurone by a gene(s) located on chromosome 6B. Euphytica.

[CR98] Mrva K, Mares D (2001). Quantitative trait locus analysis of late maturity α-amylase in wheat using doubled haploid population cranbrook × halberd. Crop Pasture Sci.

[CR99] Mrva K, Mares D (2001). Induction of late maturity a-amylase in wheat by cool temperature. Crop Pasture Sci.

[CR100] Mrva K, Mares D (2002). Screening methods and identification of QTLs associated with late maturity α-amylase in wheat. Euphytica.

[CR101] Mrva K, Wallwork M, Mares DJ (2006). Alpha-amylase and programmed cell death in aleurone of ripening wheat grains. J Exp Bot.

[CR102] Mrva K, Cheong J, Yu B (2009). Late maturity α-amylase in synthetic hexaploid wheat. Euphytica.

[CR103] Nelson SK, Steber CM (2016). Gibberellin hormone signal perception: down-regulating DELLA repressors of plant growth and development. Ann Plant Rev.

[CR104] Neoh G, Tao K, Dieters M, Fox G (2021). Late-maturity α-amylase (LMA) testing and its methodological challenges. LWT.

[CR105] Newberry M, Zwart AB, Whan A (2018). Does late maturity alpha-amylase impact wheat baking quality?. Front Plant Sci.

[CR106] Ogawa M, Hanada A, Yamauchi Y (2003). Gibberellin biosynthesis and response during arabidopsis seed germination. Plant Cell.

[CR107] Okamoto M, Kuwahara A, Seo M (2006). CYP707A1 and CYP707A2, which encode abscisic acid 8’-hydroxylases, are indispensable for proper control of seed dormancy and germination in arabidopsis. Plant Physiol.

[CR108] Olaerts H, Courtin CM (2018). Impact of preharvest sprouting on endogenous hydrolases and technological quality of wheat and bread: a review. Compr Rev Food Sci Food Saf.

[CR109] Olszewski N, Sun T-P, Gubler F (2002). Gibberellin signaling: biosynthesis, catabolism, and response pathways. Plant Cell.

[CR110] Penfield S (2017). Seed dormancy and germination. Curr Biol.

[CR111] Peng J, Harberd NP (2002). The role of GA-mediated signaling in the control of seed germination. Curr Opin Plant Biol.

[CR112] Perten H (1964). Application of the falling number method for evaluating alpha-amylase activity. Cereal Chem.

[CR113] Rahman MN, Hangs R, Schoenau J (2020). Influence of soil temperature and moisture on micronutrient supply, plant uptake, and biomass yield of wheat, pea, and canola. J Plant Nutr.

[CR114] Ral J-P, Whan A, Larroque O (2016). Engineering high α-amylase levels in wheat grain lowers falling number but improves baking properties. Plant Biotechnol J.

[CR115] Ral J-PF, Sun M, Mathy A (2018). A biotechnological approach to directly assess the impact of elevated endogenous α-amylase on Asian, white-salted noodle quality. Starch Stärke.

[CR116] Ren T, Tang Z, Fu S (2017). Molecular cytogenetic characterization of novel wheat-rye T1RS.1BL translocation lines with high resistance to diseases and great agronomic traits. Front Plant Sci.

[CR117] Rerkasem B, Jamjod S (2004). Boron deficiency in wheat: a review. Field Crop Res.

[CR118] Rich-Griffin C, Stechemesser A, Finch J (2020). Single-cell transcriptomics: a high-resolution avenue for plant functional genomics. Trends Plant Sci.

[CR119] Ritchie S, Gilroy S (1998). Calcium-dependent protein phosphorylation may mediate the gibberellic acid response in barley aleurone. Plant Physiol.

[CR120] Ritchie S, McCubbin A, Ambrose G (1999). The sensitivity of barley aleurone tissue to gibberellin is heterogeneous and may be spatially determined. Plant Physiol.

[CR121] Rogers JC, Lanahan MB, Rogers SW (1994). The cis-acting gibberellin response complex in high pi alpha-amylase gene promoters. Requirement of a coupling element for high-level transcription. Plant Physiol.

[CR122] Schramm EC, Abellera JC, Strader LC (2010). Isolation of ABA-responsive mutants in allohexaploid bread wheat (*Triticum aestivum* L.): drawing connections to grain dormancy, preharvest sprouting, and drought tolerance. Plant Sci.

[CR123] Shaaban M (2010). Role of boron in plant nutrition and human health. Am J Plant Physiol.

[CR124] Shu K, Meng YJ, Shuai HW (2015). Dormancy and germination: How does the crop seed decide?. Plant Biol (stuttg).

[CR125] Shu K, Liu X-D, Xie Q, He Z-H (2016). Two faces of one seed: hormonal regulation of dormancy and germination. Mol Plant.

[CR126] Shuai H, Meng Y, Luo X (2016). The roles of auxin in seed dormancy and germination. Yi Chuan.

[CR127] Skriver K, Olsen FL, Rogers JC, Mundy J (1991). Cis-acting DNA elements responsive to gibberellin and its antagonist abscisic acid. Proc Natl Acad Sci USA.

[CR128] Steber CM (2017). Avoiding problems in wheat with low falling numbers. Crops Soils Am Soc Agron.

[CR129] Tahir M, Tanveer A, Hussain T (2009). Yield response of wheat (*Triticum aestivum* L.) to boron application at different growth stages. Pak J Life Soc Sci.

[CR130] Tan MK, Verbyla AP, Cullis BR (2010). Genetics of late maturity α-amylase in a doubled haploid wheat population. Crop Pasture Sci.

[CR131] Tang T, Botwright Acuña T, Spielmeyer W, Richards RA (2020). Effect of gibberellin-sensitive Rht18 and gibberellin-insensitive Rht-D1b dwarfing genes on vegetative and reproductive growth in bread wheat. J Exp Bot.

[CR132] Thibivilliers S, Libault M (2021). Plant single-cell multiomics: cracking the molecular profiles of plant cells. Trends Plant Sci.

[CR133] Toyomasu T, Usui M, Sugawara C (2015). Transcripts of two ent-copalyl diphosphate synthase genes differentially localize in rice plants according to their distinct biological roles. J Exp Bot.

[CR134] Tuan PA, Kumar R, Rehal PK (2018). Molecular mechanisms underlying abscisic acid/gibberellin balance in the control of seed dormancy and germination in cereals. Front Plant Sci.

[CR135] Tuttle KM, Martinez SA, Schramm EC (2015). Grain dormancy loss is associated with changes in ABA and GA sensitivity and hormone accumulation in bread wheat, *Triticum aestivum* (L.). Seed Sci Res.

[CR136] Ueguchi-Tanaka M, Fujisawa Y, Kobayashi M (2000). Rice dwarf mutant d1, which is defective in the α subunit of the heterotrimeric G protein, affects gibberellin signal transduction. PNAS.

[CR137] Ueguchi-Tanaka M, Ashikari M, Nakajima M (2005). Gibberellin insensitive Dwarf1 encodes a soluble receptor for gibberellin. Nature.

[CR138] Ueguchi-Tanaka M, Nakajima M, Motoyuki A, Matsuoka M (2007). Gibberellin receptor and its role in gibberellin signaling in plants. Annu Rev Plant Biol.

[CR139] Van De Velde K, Chandler PM, Van Der Straeten D, Rohde A (2017). Differential coupling of gibberellin responses by Rht-B1c suppressor alleles and Rht-B1b in wheat highlights a unique role for the DELLA n-terminus in dormancy. J Exp Bot.

[CR140] Verbyla AP, Cullis BR (2012). Multivariate whole genome average interval mapping: QTL analysis for multiple traits and/or environments. Theor Appl Genet.

[CR141] Walker-Simmons M (1987). ABA levels and sensitivity in developing wheat embryos of sprouting resistant and susceptible cultivars. Plant Physiol.

[CR142] Walkowiak S, Gao L, Monat C (2020). Multiple wheat genomes reveal global variation in modern breeding. Nature.

[CR143] Wu Y, Zhou K, Toyomasu T (2012). Functional characterization of wheat copalyl diphosphate synthases sheds light on the early evolution of labdane-related diterpenoid metabolism in the cereals. Phytochemistry.

[CR144] Yamauchi Y, Ogawa M, Kuwahara A (2004). Activation of gibberellin biosynthesis and response pathways by low temperature during imbibition of *Arabidopsis thaliana* seeds. Plant Cell.

[CR145] Yan J, Zhang N, Wang X, Zhang S (2020). Differences in the physiological responses of Rht13 and rht wheat lines to short-term osmotic stress. Cereal Res Commun.

[CR146] Young TE, Gallie DR, DeMason DA (1997). Ethylene-mediated programmed cell death during maize endosperm development of wild-type and *shrunken2* genotypes. Plant Physiol.

[CR147] Zentella R, Yamauchi D, Ho TD (2002). Molecular dissection of the gibberellin/abscisic acid signaling pathways by transiently expressed RNA interference in barley aleurone cells. Plant Cell.

[CR148] Zhang Q, Li C (2017). Comparisons of copy number, genomic structure, and conserved motifs for α-amylase genes from barley, rice, and wheat. Front Plant Sci.

[CR149] Zhang J, Chen J, Bowman BC (2014). Association mapping of hagberg falling number in hard white spring wheat. Crop Sci.

[CR150] Zhang Y, Liu H, Yan G (2021). Characterization of near-isogenic lines confirmed QTL and revealed candidate genes for plant height and yield-related traits in common wheat. Mol Breed.

[CR151] Zhu J-K (2016). abiotic stress signaling and responses in plants. Cell.

[CR152] Zhu T, Wang L, Rimbert H (2021). Optical maps refine the bread wheat *Triticum aestivum* cv. Chinese spring genome assembly. Plant J.

